# Antiepileptic Drug Use and the Risk of Stroke Among Community‐Dwelling People With Alzheimer Disease: A Matched Cohort Study

**DOI:** 10.1161/JAHA.118.009742

**Published:** 2018-09-15

**Authors:** Tatyana Sarycheva, Piia Lavikainen, Heidi Taipale, Jari Tiihonen, Antti Tanskanen, Sirpa Hartikainen, Anna‐Maija Tolppanen

**Affiliations:** ^1^ School of Pharmacy Faculty of Health Sciences University of Eastern Finland Kuopio Finland; ^2^ Kuopio Research Centre of Geriatric Care University of Eastern Finland Kuopio Finland; ^3^ Department of Clinical Neuroscience Karolinska Institutet Stockholm Sweden; ^4^ Department of Forensic Psychiatry Niuvanniemi Hospital University of Eastern Finland Kuopio Finland

**Keywords:** Alzheimer disease, antiepileptic agent, stroke, Cerebrovascular Disease/Stroke, Cognitive Impairment

## Abstract

**Background:**

People with Alzheimer disease (AD) are more predisposed to seizures than older people in general, and use of antiepileptic drugs (AEDs) is more frequent. AED use has been linked to a higher risk of vascular events in the general population; however, it is not evident whether the same risk exists in people with AD. We assessed the risk of stroke associated with incident AED use among people with AD.

**Methods and Results:**

The MEDALZ (Medication Use and Alzheimer's Disease) cohort includes all Finnish people who received a clinically verified AD diagnosis (N=70718) from 2005 to 2011. People with previous strokes were excluded. For each incident AED user (n=5617) one nonuser was matched according to sex, age, and time since AD diagnosis. Analyses were conducted with Cox proportional hazards models and inverse probability of treatment weighting. Compared with nonuse, AED use was associated with an increased risk of stroke (inverse probability of treatment weighting hazard ratio (HR), 1.37; 95% confidence interval [CI], 1.07–1.74). The risk was strongest during the first 90 days (adjusted HR, 2.36; 95% CI, 1.25–4.47) of AED use. According to stroke type, the association was with ischemic strokes (inverse probability of treatment weighting HR, 1.34; 95% CI, 1.00–1.79) and hemorrhagic ones (inverse probability of treatment weighting HR, 1.44; 95% CI, 0.86–2.43). The stroke risk of users of older AEDs did not differ from that of the users of newer AEDs (adjusted HR, 1.04; 95% CI, 0.71–1.53).

**Conclusions:**

AED use was related to an increased risk of stroke, regardless of AED type. Our results highlight caution in AED use in this vulnerable population.

**Clinical Perspective**

**What Is New?**

- By comparing incident antiepileptic drug (AED) users with nonusers in the cohort of people with Alzheimer disease, we found that AED use was associated with increased risk of stroke in the proximity of the ischemic type of stroke.
- The number of incident strokes was highest during the first 180 days since the AED use initiation, with a more than twofold increase during the first 3 months of AED use.
- Users of older AEDs did not differ in risk of stroke from users of newer AEDs, and there were no differences of stroke risk between individual AEDs.

**What Are the Clinical Implications?**

- This study provides unique information on AED use associated adverse events as stroke in people with Alzheimer disease, as they are often excluded from randomized controlled trials because of comorbidities and concomitant drug use.
- The pathological changes in Alzheimer disease may increase the susceptibility to the adverse events of AEDs, and careful clinical consideration is needed before prescribing them to a person with Alzheimer disease.

## Introduction

Alzheimer disease (AD) and cerebrovascular disease share pathophysiological mechanisms and are among the leading causes of death in the older general population.^1^ People with AD are more likely to experience stroke than those without AD.^2^, ^3^ This may be due to higher prevalence of vascular risk factors or drugs acting on the central nervous system, which are more commonly used by people with AD.

Previous studies on the association between AEDs use and risk of stroke have been inconsistent. Two studies found no evidence of increased stroke risk among incident AED users in the general population.^4^, ^5^ The risk of stroke may also differ among AEDs. In the Danish cohort study among AED users with epilepsy, oxcarbazepine users had a higher risk of ischemic stroke, and valproic acid and lamotrigine users had a lower risk in comparison with carbamazepine users^6^ In another study, the risk of ischemic stroke was lower among valproic acid users than those who used other AEDs^7^

None of these previous studies assessed the stroke risk in older people or people with AD, although they use AEDs relatively often. In studies of both community‐dwelling and nursing home residents, the prevalence of AED use varied between 2% and 10% among older people.^8^, ^9^, ^10^, ^11^ It has been shown that community dwellers with AD use AEDs more often than those without AD and that they also use older AEDs more frequently.^12^

Thus, we investigated whether incident AED use was associated with higher risk of strokes in people with AD. We also assessed the risk of ischemic and hemorrhagic strokes separately and performed drug‐drug comparisons.

## Materials and Methods

### Data Availability

The data used to conduct the research are available from the corresponding author, but restrictions by the register maintainers and Finnish legislation apply to the availability of these data. Therefore, the data are not publicly available. However, data are available from the authors upon reasonable request and with permission of the register maintainers.

### Study Population and Data Sources

This study was based on a nationwide register‐based MEDALZ (Medication Use and Alzheimer's Disease) cohort. The MEDALZ cohort includes all community‐dwelling people who received diagnoses of AD in Finland from 2005 to 2011 (N=70 718).^13^ People with an AD diagnosis were identified from the Special Reimbursement Register, which contains records of people who are entitled to higher medication reimbursement because of chronic diseases, including AD. All citizens and long‐term residents of Finland are covered under the Finnish National Health Insurance scheme and are thus eligible for reimbursement of medical expenses under the Health Insurance Act. To be entitled to a special reimbursement because of a chronic disease, a patient must meet predefined criteria, and a diagnosis statement must be submitted to the Social Insurance Institution of Finland (SII) for approval. For AD, the SII requires that the medical statement verifies that the patient has (1) symptoms consistent with AD; (2) experienced a decrease in social capacity over a period of at least 3 months; (3) received a computed tomography/magnetic resonance imaging scan; (4) had possible alternative diagnoses excluded; and (5) received confirmation of the diagnosis by a registered geriatrician or neurologist. The SII reviews all medical statements and grants special reimbursement if the criteria are fulfilled. The diagnosis of AD is based on the National Institute of Neurological and Communicative Disorders and Stroke and the Alzheimer's Disease and Related Disorders Association^14^ and *Diagnostic and Statistical Manual of Mental Disorders,* Fourth Edition, criteria for AD.

Data on purchased drugs since 1995 were extracted from the Prescription register maintained by the SII. This register contains records of all reimbursed drug purchases made by all Finnish community‐dwelling residents and includes the dispensing date of each prescription, the World Health Organization Anatomical Therapeutic Chemical code, number of dispensed packages, and number of tablets dispensed. The information on the drug use during hospitalization/institutionalization is not recorded in the Prescription register; therefore, people hospitalized or institutionalized for more than 182 days during the washout period or >90 days at the end of the washout period were excluded. Dates of long‐term institutionalization were obtained from the SII and durations of hospital stays from the Care Register for Health Care. Incident strokes were identified from the Care Register for Health Care and Causes of Death register. Data on hospitalization‐based confounders were retrieved from the Care Register for Health Care.

Only people who initiated AED use after AD diagnosis were included in this study. A 1‐year washout period before AED initiation was applied to identify incident use during the study period. People who had used AEDs during this period were excluded to avoid prevalent user bias.^15^ We excluded people who had acute cancer (definition explained in Table S1) during the washout period or previous stroke before the follow‐up. Incident strokes, unlike prevalent ones, can be reliably identified with the applied registers.^16^ Formation of the study sample is described in the Figure.

**Figure 1:**
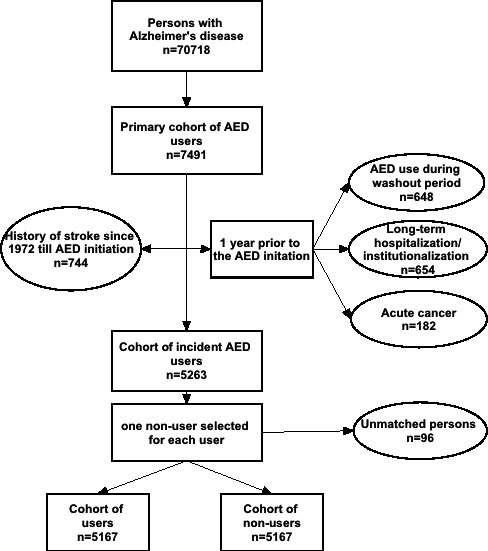
Flowchart of exclusion criteria of the study cohort with Alzheimer disease diagnoses.

A matched nonuser was identified for each user with the same inclusion/exclusion criteria (Figure), applying incidence density sampling without replacement. The matching criteria were age (±730 days), sex, and time since AD diagnosis (±90 days). People without a match (n=96) were excluded from further analyses. The follow‐up started on the index date, which was the date of AED initiation or the corresponding matching date for nonusers. People were followed until the incidence of stroke, death, AED use discontinuation (for users), AED initiation (for nonusers), continuous hospitalization/institutionalization lasting >90 days, after 3 years of follow‐up, or the end of the study (December 31, 2015). In drug‐drug comparisons, the follow‐up also ended if there were switches between AEDs or polytherapy was initiated.

According to Finnish legislation, no ethics committee approval was required for this study because only deidentified register‐based data were used and the study participants were not contacted. The study was approved by the register maintainers.

### Antiepileptic Drug Use Exposure

AEDs were categorized as older and newer according to previous classifications.^17^ Older AEDs included valproic acid, carbamazepine, clonazepam, and phenytoin; newer AEDs included pregabalin, gabapentin, lamotrigine, oxcarbazepine, topiramate, and levetiracetam. Use of only 1 AED was considered as a monotherapy and concomitant use of >1 AED, regardless of the category, as a polytherapy.

AED purchases were modeled to use periods for each person and for each Anatomical Therapeutic Chemical code during the follow‐up with a validated PRE2DUP (from prescription drug purchases to drug use periods) method^18^ In brief, based on each person's purchase history for each Anatomical Therapeutic Chemical code, this method constructs continuous drug use periods, calculates a sliding average of daily dose in defined daily doses, and combines purchases of the same drug by taking into account stockpiling, purchase regularity, dose changes, and periods of hospitalization/institutionalization^18^, ^19^ After modeling each drug substance, overlapping periods of AEDs were combined to retrieve time when any AED was used for use versus no‐use comparisons^18^ and similarly time on old versus new AED use.

### Outcome

The main outcome measure included all strokes treated in hospitals and all strokes confirmed as direct or underlying cause of death. We restricted the study to incident strokes; thus, only the first stroke after the start of follow‐up for each individual was considered. Incident strokes were identified with *International Classification of Diseases, Tenth Revision* (*ICD‐10*) codes I60 to I64 and further divided into ischemic strokes (I63), hemorrhagic strokes (I60–I62), and unspecified strokes (I64) for additional subtype analyses. Unspecified strokes were not analyzed separately because of the small number of events in the data set. Previous strokes were identified also according to corresponding *International Classification of Diseases, Eighth Revision* (*ICD‐8*) and *Ninth Revision* (*ICD‐9*) codes.

### Confounders

Data on hospitalization‐based confounders since 1994 until the index date were retrieved on the basis of *ICD‐10* codes, whereas those based on entitlements to higher special reimbursements were defined as occurring ever after establishment of the Special Reimbursement register in 1972 until the index date.

From these registers we identified the following cardiovascular comorbidities: ischemic heart disease, cardiac arrhythmias, hypertension, chronic heart failure, and peripheral artery disease.

In addition, we considered mental comorbidities, such as schizophrenia, depression, or bipolar disorders, and substance abuse as confounders. Schizophrenia diagnoses were restricted to those that were diagnosed at least 5 years before AD diagnosis based on possible misclassification caused by the rapid development of AD symptoms after this date^20^ Substance abuse was defined as alcohol‐induced chronic pancreatitis, mental and behavioral disorders caused by psychoactive substance abuse, and/or substance abuse as a reason for admission.

Other comorbidities included diabetes mellitus, asthma or chronic obstructive pulmonary disease, rheumatoid arthritis, epilepsy, head trauma, and hip fracture.

Use of antipsychotics, benzodiazepines and related drugs, antidepressants, nonsteroidal anti‐inflammatory drugs, and antithrombotic agents within a 1‐year period before the index date were identified from the PRE2DUP modeled drug use data.

Detailed definitions and classifications of confounders are provided in Table S1.

### Statistical Analyses

Descriptive statistics are presented as means with standard deviations or frequencies with proportions.

The risk of stroke was compared between AED users and nonusers applying Cox proportional hazards regression models. We used a robust variance estimator in the models for AED user and nonuser comparisons to account for the matched nature of our sample. Proportional hazards assumptions were confirmed by exploring parallelism of log‐negative and log estimated survival curves for each covariate. Hazard ratios (HRs) with their corresponding 95% confidence intervals (CIs) were estimated. First, we produced a crude, unadjusted HR for the effect of AED use on stroke risk. Second, the model was adjusted for age; sex; use of antidepressants, antipsychotics, benzodiazepines and related drugs, antithrombotic agents, and nonsteroidal anti‐inflammatory drugs; and history of hypertension, ischemic heart disease, chronic heart failure, cardiac arrhythmia, peripheral artery disease, diabetes mellitus, epilepsy, and head trauma.

Third, to better control for confounding by indication and balance potential confounders between the comparison groups, we weighted the Cox model with inverse probability of treatment (IPT) weights based on propensity score. Selection of variables (Table S1) for the IPT weights was based on their potential association with the outcome and the exposure. We estimated the propensity score with a logistic regression model as the conditional probability of AED use conditioned on the covariates measured at the baseline. Balancing properties of the IPT weighting between the AED users and matched nonusers were ascertained by comparing covariate distributions before and after IPT weighting using the standardized difference. A standardized difference >10% was considered an indication of a meaningful difference.^21^ We also performed these analyses in relation to type of stroke.

Subsequently, we quantified the risk of stroke according to the duration of AED use by reformulating the data into a counting process format. We divided duration of AED use into 4 periods; 1 to 90 days, 91 to 180 days, 181 to 365 days, and 366 to 1095 days. Then, all 3 previous Cox models were conducted separately for each time period.

In analyses comparing individual AEDs (user‐only design), risk of stroke was compared between the most frequently used AEDs, where users of multiple AEDs were excluded (n=30). Valproic acid was chosen as a reference AED.

Finally, we applied Fine & Gray subdistribution hazard models^22^ to account for the effect of competing risk of death attributable to other causes on our primary results. Thus, we examined the instantaneous rate of stroke in subjects who are still alive or who have previously died from causes other than stroke.

All analyses were performed with SAS (Version 9.4; SAS Institute Inc, Cary., NC).

## Results

Altogether, 7491 individuals initiated AED use during the follow‐up, of whom 5167 met the inclusion criteria (Figure). Compared with nonusers, AED users more often had chronic cardiovascular diseases and mental and behavioral disorders such as depression and bipolar disorders; almost all diagnoses of epilepsy were observed among them (Table S2). AED users were also more likely to use antidepressants, antipsychotics, benzodiazepines and related drugs, and nonsteroidal anti‐inflammatory drugs.

Differences within the individual antiepileptic medications are described in Table S3.

Mean follow‐up time was shorter among users compared with nonusers (351.7 days versus 728.6 days, respectively). AED discontinuation (54.7%) was the most common reason for censoring among AED users, whereas nonusers censored most often after 3 years of follow‐up (39.3%).

In total, 328 people experienced a stroke during the follow‐up, yielding incidence rates of 2.75 (95% CI, 2.33–3.26) per 100 person‐years among users and 1.85 (95% CI, 1.61–2.14) per 100 person‐years among nonusers (Table 1). After applying IPT weighting, AED use was associated with a 37% increased relative risk of stroke compared with nonuse (IPT weighting HR, 1.37; 95% CI, 1.07–1.74). When the analyses were stratified by stroke type, AED use was related to an increased risk of ischemic strokes (IPT weighting HR, 1.34; 95% CI, 1.00–1.79), while the confidence intervals for hemorrhagic strokes were wide (IPT weighting HR, 1.44; 95% CI, 0.86–2.43). In the secondary analyses adjusted for competing risk of death attributable to other causes, the associations between AED use and all strokes (subdistribution HR, 1.22; 95% CI; 0.95–1.55) as well as ischemic strokes (subdistribution HR, 1.25; 95% CI, 0.94–1.65) were weaker than in the primary analyses.

**Table 1:** Association Between Antiepileptic Drug Use and Stroke Overall and Stratified by Types of Stroke

	No. of People	No. of Strokes	Person‐Years of Follow‐Up	Strokes per 100 Person‐Years (95% CI)	Unadjusted HR (95% CI)	Adjusted HR^a^ (95% CI)	IPT Weighted HR^b^ (95% CI)	Subdistribution HR^c^ (95% CI)
All strokes								
Nonusers	5167	191	10 307	1.85 (1.61–2.14)	1.00	1.00	1.00	1.00
AED users	5167	137	4975	2.75 (2.33–3.26)	1.45 (1.17–1.80)	1.31 (1.03–1.68)	1.37 (1.07–1.74)	1.22 (0.95–1.55)
Types of stroke								
Ischemic strokes								
Nonusers	5167	143	10 307	1.39 (1.18–1.63)	1.00	1.00	1.00	1.00
AED users	5167	104	4975	2.09 (1.72–2.53)	1.45 (1.12–1.87)	1.30 (0.97–1.74)	1.34 (1.00–1.79)	1.25 (0.94–1.65)
Hemorrhagic strokes								
Nonusers	5167	41	10 307	0.40 (0.29–0.54)	1.00	1.00	1.00	1.00
AED users	5167	30	4975	0.60 (0.42–0.86)	1.41 (0.87–2.27)	1.33 (0.76–2.30)	1.44 (0.86–2.43)	1.19 (0.69–2.03)
Unspecified strokes								
Nonusers	5167	9	10 307	0.09 (0.05–0.17)	1.00	1.00	1.00	1.00
AED users	5167	3	4975	0.06 (0.02–0.19)	0.47 (0.12–1.91)	0.48 (0.12–1.93)	0.45 (0.11–1.84)	0.61 (0.17–2.24)

AED indicates antiepileptic drug; CI, confidence interval; HR, hazard ratio; IPT, inverse probability of treatment.

a Adjusted for age; sex; use of antidepressants, antipsychotics, benzodiazepines and related drugs, antithrombotic agents, and nonsteroidal anti‐inflammatory drugs; and history of hypertension, ischemic heart disease, chronic heart failure, cardiac arrhythmia, peripheral vascular disease, diabetes mellitus, epilepsy, and head trauma.

b Adjusted for baseline confounders presented in Table S1.

c Adjusted Fine & Gray subdistribution hazard model accounting for competing risk of death attributable to other causes.

In the analyses accounting for the duration of treatment (Table 2), the association between AED use and stroke was strongest during the first 90 days after AED initiation (adjusted HR, 2.36; 95% CI, 1.25–4.47), diminished after the first 90 days (adjusted HR for 6 months’ use, 1.80; 95% CI, 1.00–3.24) and disappeared after that. Accounting for competing risk of death attributable to other causes did not alter the estimation of results.

**Table 2:** Association Between Antiepileptic Drug Use and Stokes Stratified by Follow‐Up Time

	No. of People	No. of Strokes	Person‐Years of Follow‐Up	Strokes per 100 Person‐Years (95% CI)	Unadjusted HR (95% CI)	Adjusted HR^a^ (95% CI)	Subdistribution HR^b^ (95% CI)
Duration of follow‐up							
1–90 d							
Nonusers	5167	16	1232	1.30 (0.80–2.12)	1.00	1.00	1.00
AED users	5167	38	1023	3.71 (2.70–5.11)	2.87 (1.60–5.15)	2.36 (1.25–4.47)	2.31 (1.22–4.36)
91–80 d							
Nonusers	4823	28	1149	2.44 (1.68–3.53)	1.00	1.00	1.00
AED users	3288	29	714	4.06 (2.82–5.84)	1.69 (1.01–2.84)	1.80 (1.00–3.24)	1.79 (1.00–3.22)
181–365 d							
Nonusers	4504	34	2121	1.60 (1.14–2.24)	1.00	1.00	1.00
AED users	2584	22	1092	2.01 (1.33–3.06)	1.26 (0.73–2.15)	1.22 (0.68–2.18)	1.22 (0.69–2.19)
366–1095 d							
Nonusers	3902	113	5805	1.95 (1.62‐–2.34)	1.00	1.00	1.00
AED users	1806	48	2146	2.24 (1.69–2.97)	1.13 (0.81–1.58)	0.98 (0.66–1.44)	0.98 (0.67–1.44)

AED indicates antiepileptic drug; CI, confidence interval; HR, hazard ratio.

a Adjusted for age; sex; use of antidepressants, antipsychotics, benzodiazepines and related drugs, antithrombotic agents, and nonsteroidal anti‐inflammatory drugs; and history of hypertension, ischemic heart disease, chronic heart failure, cardiac arrhythmia, peripheral vascular disease, diabetes mellitus, epilepsy, and head trauma.

b Adjusted Fine & Gray subdistribution hazard model accounting for competing risk of death attributable to other causes.

Users of older AEDs did not differ in risk or relative incidence of stroke from users of newer AEDs. There were no statistically significant differences of stroke risk among individual AEDs (Table 3).

**Table 3:** Association Between Type of Antiepileptic Drug and Stroke by Type of AED

Type of AED	No. of People	No. of Strokes	Person‐Years of Follow‐Up	Strokes per 100 Person‐Years (95% CI)	Unadjusted HR (95% CI)	Adjusted HR^a^ (95% CI)	Subdistribution HR^b^ (95% CI)
Valproic acid	1582	41	1404	2.92 (2.15–3.97)	1.00	1.00	1.00
Pregabalin	2291	55	2045	2.69 (2.06–3.50)	0.95 (0.64–1.43)	0.89 (0.56–1.41)	1.06 (0.67–1.67)
Carbamazepine	297	12	254	4.72 (2.68–8.32)	1.68 (0.88–3.20)	1.50 (0.78–2.89)	1.44 (0.76–2.75)
Clonazepam	256	4	181	2.21 (0.83–5.89)	0.76 (0.27–2.13)	0.85 (0.30–2.42)	0.94 (0.33–2.65)
Oxcarbazepine	214	13	230	5.65 (3.28–9.73)	2.10 (1.12–3.93)	1.80 (0.95–3.42)	1.62 (0.84–3.13)
Gabapentin	307	5	239	2.09 (0.87–5.03)	0.74 (0.29–1.87)	0.61 (0.23–1.58)	0.71 (0.27–1.84)
Phenytoin	92	1	109	0.92 (0.13–6.51)	0.34 (0.05–2.46)	0.32 (0.04–2.37)	0.33 (0.05–2.43)
Other AEDs^c^	98	6	100	6.00 (2.70–13.35)	2.15 (0.91–5.07)	1.88 (0.79–4.46)	1.82 (0.76–4.39)
							
Newer AEDs	2808	71	2592	2.74 (2.17–3.46)	1.00	1.00	1.00
Older AEDs	2121	54	1947	2.77 (2.12–3.62)	0.99 (0.70–1.41)	1.04 (0.71–1.53)	0.95 (0.65–1.39)
Both newer and older AEDs	238	12	437	2.75 (1.56–4.83)	1.06 (0.57–1.96)	1.02 (0.54–1.94)	1.05 (0.55–2.02)

AED indicates antiepileptic drug; CI, confidence interval; HR, hazard ratio.

a Adjusted for age; sex; use of antidepressants, antipsychotics, benzodiazepines and related drugs, antithrombotic agents, and nonsteroidal anti‐inflammatory drugs; and history of hypertension, ischemic heart disease, chronic heart failure, cardiac arrhythmia, peripheral vascular disease, diabetes mellitus, epilepsy, and head trauma.

b Adjusted Fine & Gray subdistribution hazard model accounting for competing risk of death attributable to other causes.

c The group includes users of primidone (n=5), lamotrigine (n=26), topiramate (n=7), and levetiracetam (n=60). Users initiating with polypharmacy were excluded (n=30).

## Discussion

In this study among people with AD, AED users had a 37% higher relative risk of any stroke than nonusers, and there were no differences among individual AEDs or between older and newer AEDs. The association was more evident with ischemic strokes than hemorrhagic ones partly because of the low number of hemorrhagic strokes in our cohort.

The findings with ischemic strokes are in accordance with a previous study, although the risk increase was much lower in our study.^6^ This might be explained by the fact that only a minority of AED users had an epilepsy diagnosis in our cohort and by the difference in methods, where they assessed drug use at 2 time points rather than as a continuous exposure, which makes their results questionable. According to previous studies,^23^, ^24^, ^25^ some AEDs (eg, phenytoin and carbamazepine) seem to induce atherogenic serum cholesterol levels and thus can accelerate atherosclerosis and elevate the risk of cerebrovascular events. However, in our data, the most commonly used AEDs were valproic acid and pregabalin, and they are not known to induce atherosclerosis.

In our study, the number of strokes per 100 person‐years was highest during the first 180 days of AED use, and the relative risk was more than twofold during the first 90 days compared with nonusers. The strong association between the acute exposure and risk of stroke may be explained by protopathic bias. Some studies suggested that nondiagnosed seizures might increase the risk for subsequent stroke.^26^, ^27^ Several AEDs (eg, valproic acid, carbamazepine) are used in seizure control and, therefore, might be initiated for controlling symptoms of subsequent stroke. However, this does not explain our findings completely, as pregabalin was the most commonly used AED in our cohort and it is mainly used for chronic pain^28^ The lack of association after 6 months of AED use may also be attributable to the fact that almost one third of people within this period discontinued AED use in our cohort. Similar short duration of AED use was also observed in a study of old people with AEDs for new‐onset epilepsy.^29^ In that study, about 40% of people had a diagnosis of dementia or some psychiatric disorder, and ≈50% were nonadherent to AED treatment within a year. In addition, Zaccara et al, in a meta‐analysis of placebo‐controlled trials, investigated differences in short‐term tolerability and adverse event profile across newer AEDs and found that pregabalin, the most frequently used AED in our cohort, was among the AEDs with a higher risk for intolerable adverse effects^30^, ^31^

Concerning differences between AEDs, a previous study reported that oxcarbazepine users had a higher risk of ischemic stroke and valproic acid and lamotrigine users had a lower risk in comparison with carbamazepine users among people with epilepsy^6^ In our study with valproic acid as a reference, carbamazepine and oxcarbazepine were suggestive of an increased risk of stroke, although the results did not reach statistical significance. However, the number of users in a comparison of individual AEDs in our cohort was too small for investigations with sufficient statistical power. Therefore, more studies should be conducted on the association among individual AEDs and risk of stroke.

### Strengths and Limitations

A strength of this study is our cohort that is nationally representative and included all community dwellers diagnosed with AD. The Finnish Special Reimbursement register covers all citizens, and the accuracy of AD diagnosis in the register has been validated previously^32^ People with a mixed form of dementia (AD with vascular lesions or AD with signs of Lewy body dementia) are also eligible for special reimbursement, and thus our study also includes people with mixed dementia where AD is one counterpart. However, people with only vascular dementia were not included. As our study was restricted to people who were community dwelling at the time of AD diagnosis, the results may not be generalizable to institutionalized people. We excluded institutionalized people, as their drug exposure status could not be reliably ascertained^32^ For the same reason, long hospital stays were used as an exclusion criteria and as a reason for ending the follow‐up to avoid misclassification of the AED exposure. We excluded people with previous AED use (during 1 year) to avoid prevalent user bias, because people who had stayed on AED treatment for a longer time before the follow‐up were more likely to tolerate the drug better and their inclusion would have biased the results.

We also used the data on purchased drugs instead of self‐ or proxy‐reported data, or data on merely prescribed drugs. Self‐reported drug use is subjective to recall bias. AED use was modeled with the PRE2DUP method, which has been shown to have good validity for regularly used medications^19^ However, our data lack indications of drug use as well as symptoms and severity of AD.

We limited the study to incident strokes to avoid bias caused by possible impairments persisting from past stroke and their possible impacts on drug use^15^ Although the validity of stroke diagnoses in the Care Register for Health Care is high^16^ there is a risk that some cases of stroke were misdiagnosed or undiagnosed among people with AD and, therefore, not included in our analyses. Nondifferential diagnostic misclassification of outcome might potentially mask a true association and lead to underestimation, but it would not affect the point estimate. Another limitation of the register‐based data source is a lack of information on lifestyle factors such as smoking, body mass index, and nutrition. Exclusion of people with a history of previous stroke, extensive covariate adjustment, accounting for the matched nature of our sample, IPT weighting of models and analyses for competing risk of death attributable to other causes in our design were used to minimize these limitations and residual confounding.

As some AEDs have been linked to accelerated atherosclerosis^23^, ^24^, ^25^ one possibility would have been to assess major adverse cardiovascular events as an outcome. We limited our study to stroke because the aim of this study was to investigate whether AEDs as central nervous system–acting drugs are related to stroke risk in people with AD and because strokes can further accelerate cognitive deterioration in this group. Atherosclerosis is not a likely explanation for our findings because the highest number of strokes occurred during the first 90 to 180 days after the AED initiation supporting acute mechanisms, whereas the development of acute events attributable to metabolic changes requires a longer time. In addition, mainly older AEDs (carbamazepine, phenobarbital, phenytoin, and primidone) have been associated with alterations in lipid metabolism^4^, ^5^ These were among the least frequently used AEDs in our cohort.

## Conclusions

AED use was associated with an increased risk of stroke in people with AD, and the risk was highest during the first months of use. The risk of stroke was similar in older and newer AEDs. Our results highlight caution in AED use in this vulnerable population, and such possible complication as a stroke should be kept in mind while prescribing AEDs.

## Sources of Funding

Dr Tolppanen is funded by the Academy of Finland (grants 307232, which paid for Dr Sarycheva's salary, and 295334); Drs Taipale and Tolppanen acknowledge strategic funding from the University of Eastern Finland.

## Disclosures

Dr Tiihonen served as a consultant to Lundbeck, Organon, Janssen‐Cilag, Eli Lilly, AstraZeneca, F. Hoffman‐La Roche, and Bristol‐Myers Squibb. He received fees for giving expert opinions to Bristol‐Myers Squibb and GlaxoSmithKline, lecture fees from Janssen‐Cilag, Bristol‐Myers Squibb, Eli Lilly, Pfizer, Lundbeck, GlaxoSmithKline, AstraZeneca, and Novartis; and a grant from Stanley Foundation. Dr Tiihonen is a member of the advisory board in AstraZeneca, Janssen‐Cilag, and Otsuka. Dr Hartikainen received a lecturing fee from MSD. Drs Taipale, Tiihonen, and Tolppanen participated in research projects funded by Janssen and Eli Lilly, with grants paid to the institution where they were employed. Dr Tolppanen is a member of the Janssen advisory board. The remaining authors have no disclosures to report.

## Supporting information


**Table S1.** Definitions for Exclusion Criteria
**Table S2.** Characteristics of the Study Sample of People With Alzheimer Disease According to the Use of Antiepileptic Drugs (n=10 334)
**Table S3.** Characteristics of Antiepileptic Drug Users by Type of Antiepileptic Drug (n=5137*)
