# Supplementary material for: Antiepileptic Drug Use and the Risk of Stroke Among Community‐Dwelling People With Alzheimer Disease: A Matched Cohort Study
Source: J Am Heart Assoc. 2018 Sep 15;7(18):e009742. doi: 10.1161/JAHA.118.009742 (PMC6222965; doi:10.1161/JAHA.118.009742)
Supplement: Supplementary file 1 — Table S1. Definitions for Exclusion Criteria Table S2. Characteristics of the Study Sample of People With Alzheimer Disease According to the Use of Antiepileptic Drugs (n=10 334) Table S3. Characteristics of Antiepileptic Drug Users by Type of Antiepileptic Drug (n=5137*) [file JAH3-7-e009742-s001.pdf]

# SUPPLEMENTAL MATERIAL

**Table S1. Definitions for exclusion criteria.**

| Definition | ICD-code/ATC-code/other code | Measurement period | Data source |
|---|---|---|---|
| Use of antiepileptic medication | ATC: N03A | Within one year prior to the start of follow-up | PR |
| History of stroke | ICD-10: I60–I64<br>ICD-9: 430, 431, 432, 4330A, 4331A, 4339A, 4349A, 4340A, 4341A, 4360<br>ICD-8: 430, 431, 432, 433, 434 | Diagnosed since 1972 until the start of follow-up as a primary or secondary diagnosis | FCR |
| Acute cancer | ATC: L01 (excluding persons with L01BA01 and special reimbursement for rheumatoid arthritis), L02, L03AA, L03AB01, L03AB04, L03AB05, L03AC, L03AX (excluding L03AX13), L04AA10, L04AA34, L04AA18, L04AX02, L04AX03 (excluding persons with special reimbursement for rheumatoid arthritis) | Within one year prior to the start of follow-up | PR |
| Acute cancer | NOMESCO: AAG50, AX, HA0, PJO, QA0, QB0, QC0, QD0, QW0, QX0, WA, WB, WC, WD, WE, WF0, WFO, ZX0 | Within one year prior to the start of follow-up | FCR |

| | | | |
|---|---|---|---|
| Long-term hospitalization/ institutionalization | Hospitalized or institutionalized over 50% of the period or an ongoing stay of ≥90 days at the end of the period | Within one year prior to the start of follow-up | Register maintained by the SII |
| Death | | | SF |

ATC, Anatomical Therapeutic Chemical; FCR, Finnish Care Register for Health care; ICD, International Classification of Diseases; PR, Prescription Register; SF, Statistics Finland; SII, Social Insurance Institution of Finland.

**Definitions and classifications of baseline covariates.**

| **Socio-demographic and socio-economic factors** | **Classification** | **Measurement point** | **Data source** |
|---|---|---|---|
| Age, years | –64<br>65–74<br>75–84<br>85– | Baseline | PR |
| Sex | Male<br>Female | Baseline | PR |
| University hospital catchment area | Helsinki<br>Turku<br>Tampere<br>Kuopio<br>Oulu | Baseline | SF |
| Occupational socioeconomic position | Managerial/professional<br>Office<br>Farming/forestry<br>Sales, industrial, cleaning<br>Unknown and those with missing data at Statistics Finland) | Highest position recorded since 1972 - 3 years prior to the diagnosis of Alzheimer's disease | SF |
| | | | |
| **Medication use at baseline** | **ATC code** | **Measurement period** | **Data source** |
| Antidepressants | N06A | Within one year prior to the start of follow-up | PR |
| Antipsychotics | N05A excluding lithium N05AN01 | Within one year prior to the start of follow-up | PR |
| Antithrombotic agents | B01A | Within one year prior to the start of follow-up | PR |
| Benzodiazepines and related drugs | Benzodiazepines N05BA, N05CD and/or Z-drugs N05CF | Within one year prior to the start of follow-up | PR |

| | | | |
|---|---|---|---|
| Nonsteroidal anti-inflammatory drugs | M01A (excluding M01AX05) | Within one year prior to the start of follow-up | PR |
| | | | |
| **Cardiovascular comorbidities** | **ICD-10 code or Classification number** | **Measurement period** | **Data source** |
| Hypertension | Hospitalization (ICD-10: I10–I15) or special reimbursement (classification number 205) | Diagnosed since 1994 until the start of follow-up | FCR, SRR |
| Coronary artery disease | Hospitalization (ICD-10: I20–I25) or special reimbursement (classification numbers 206, 213, 280, 211) | Diagnosed since 1994 until the start of follow-up | FCR, SRR |
| Chronic heart failure | Hospitalization (ICD-10: I43–43, I50, I110) or special reimbursement (classification number 201) | Diagnosed since 1994 until the start of follow-up | FCR, SRR |
| Cardiac arrhythmia | Hospitalization (ICD-10: I46–I49) or special reimbursement (classification number 207) | Diagnosed since 1994 until the start of follow-up | FCR, SRR |
| Peripheral arterial disease | Hospitalization (ICD-10: I70, I71.2, I71.4, I71.6, I71.9, I73, I77, I79, K55.1, K55.9, Z95.8) | Diagnosed since 1994 until the start of follow-up | FCR |
| | | | |
| **Mental comorbidities** | **ICD-10 code** | **Measurement period** | **Data source** |
| Schizophrenia | Hospitalization (ICD-10: F20–F29 (schizophrenia, schizotypal or delusional disorders)) | Diagnosed since 1994 until 5 years prior to the diagnosis of AD | FCR |
| Depression or bipolar disorder | Hospitalization (ICD-10: F30–F34, F38–F39) | Diagnosed since 1994 until the start of follow-up | FCR |

| | | | |
|---|---|---|---|
| Substance abuse | Hospitalization (ICD-10: K86.0 (alcohol-induced chronic pancreatitis), F10–19 (mental and behavioral disorders due to psychoactive substance abuse) and/or substance abuse as reason for admission) | Diagnosed since 1994 until the start of follow-up | FCR |
| | | | |
| **Other comorbidities** | **ATC-code, ICD-10 code or Classification number** | **Measurement period** | **Data source** |
| Diabetes | Diabetes medication (ATC: A10), hospitalization (ICD-10: E10–E14, E89.1) or special reimbursement for diabetes (classification number 103) | Within one year prior to the start of follow-up in the PR or diagnosed since 1994 in the FCR or since 1972 until the start of follow-up in the SRR | PR, FCR, SRR |
| Asthma/COPD | Hospitalization (ICD-10: I26, J41, J42, J44, J439, J45, J47) or special reimbursement (classification numbers 203, 210) | Diagnosed since 1994 until the start of follow-up in the FCR or since 1972 until the start of follow-up in the SRR | FCR, SRR |
| Rheumatoid arthritis | Hospitalization (ICD-10: M05, M06, M45) or special reimbursement (classification number 202) | Diagnosed since 1994 until the start of follow-up in the FCR or since 1972 until the start of follow-up in the SRR | FCR, SRR |
| Epilepsy | Special reimbursement (classification number 111) | Diagnosed since 1972 until the start of follow-up | SRR |
| Head trauma | Hospitalization (ICD-10: S0* (Injuries to the head)) | Diagnosed since 1994 until the start of follow-up | FCR |
| Hip fracture | Hospitalization (ICD-10: S72.0–S72.2) | Diagnosed since 1994 until the start of follow-up | FCR |

AD, Alzheimer’s disease; ATC, Anatomical Therapeutic Chemical; COPD, chronic obstructive pulmonary disease; FCR, Finnish Care Register; ICD, International Classification of Diseases; PR, Prescription Register; SF, Statistics Finland; SRR, Special Reimbursement Register.

**Table S2**. **Characteristics of the study sample of persons with Alzheimer's disease according to the use of antiepileptic drugs (n=10 334).**

| Variables | AED users (n=5 167) | | Non-users (n=5 167) | | Unweighted Standardized Difference | IPT weighted Standardized Difference |
|---|---|---|---|---|---|---|
| Age (years), mean(SD) | 80.7 | 7.62 | 80.7 | 7.54 | 0.4 | 0.1 |
| Age (years), n(%) | | | | | | |
| *< 65* | 214 | 4.1 | 211 | 4.1 | 0.3 | 2.5 |
| *65–74* | 830 | 16.1 | 806 | 15.6 | 1.3 | 2.7 |
| *75–84* | 2 543 | 49.2 | 2 568 | 49.7 | 1.0 | 0.3 |
| *≥ 85* | 1 580 | 30.6 | 1 582 | 30.6 | 0.1 | 0.6 |
| Women, n(%) | 3 468 | 67.1 | 3 468 | 67.1 | 0.1 | 0.6 |
| Time since AD diagnosis*, mean(SD) | 1 021.9 | 801.6 | 1 022.2 | 800.7 | 0.0 | 1.0 |
| University hospital catchment area, n(%) | | | | | | |
| *Helsinki* | 1 617 | 31.3 | 1 390 | 26.9 | 9.7 | 2.3 |
| *Turku* | 686 | 13.3 | 651 | 12.6 | 2.0 | 0.6 |
| *Tampere* | 1 058 | 20.5 | 1 304 | 25.2 | 11.4 | 1.3 |
| *Kuopio* | 912 | 17.7 | 1 037 | 20.1 | 6.2 | 4.7 |
| *Oulu* | 894 | 17.3 | 785 | 15.2 | 5.7 | 0.4 |
| Occupational status†, n(%) | | | | | | |
| *Managerial/professional* | 2 244 | 43.4 | 2 158 | 41.8 | 3.4 | 3.7 |
| *Office* | 838 | 16.2 | 919 | 17.8 | 4.2 | 2.0 |
| *Farming/forestry* | 440 | 8.5 | 480 | 9.3 | 2.7 | 1.4 |
| *Sales, industrial, cleaning* | 1 200 | 23.2 | 1 185 | 22.9 | 0.7 | 1.8 |
| *Unknown, no response* | 445 | 8.6 | 425 | 8.2 | 1.4 | 0.2 |
| **Medication use, n(%)** | | | | | | |
| *Antidepressants* | 2 228 | 43.1 | 1 488 | 28.8 | 30.2 | 2.9 |
| *Antipsychotics* | 1 968 | 38.1 | 1 192 | 23.1 | 33.0 | 3.4 |
| *Antithrombotic agents* | 1 330 | 25.7 | 1 157 | 22.4 | 7.8 | 0.1 |
| *Benzodiazepines and related drugs* | 2 324 | 45.0 | 1 343 | 26.0 | 40.5 | 2.2 |
| *Nonsteroidal anti-inflammatory drugs* | 1 224 | 23.7 | 701 | 13.6 | 26.2 | 2.0 |
| **Comorbidities, n(%)** | | | | | | |
| ***Cardiovascular diseases*** | | | | | | |
| *Hypertension* | 2 305 | 44.6 | 2 121 | 41.1 | 7.2 | 1.3 |
| *Ischemic heart disease* | 1 511 | 29.2 | 1 347 | 26.1 | 7.1 | 4.1 |
| *Chronic heart failure* | 681 | 13.2 | 640 | 12.4 | 2.4 | 0.2 |
| *Cardiac arrhythmia* | 901 | 17.4 | 821 | 15.9 | 4.2 | 2.3 |
| *Peripheral vascular disease* | 180 | 3.5 | 112 | 2.2 | 7.9 | 2.5 |
| ***Mental diseases*** | | | | | | |
| *Schizophrenia* | 34 | 0.7 | 27 | 0.5 | 1.8 | 0.7 |
| *Depression or bipolar disorder* | 307 | 5.9 | 177 | 3.4 | 11.9 | 1.4 |
| *Substance abuse* | 166 | 3.2 | 106 | 2.1 | 7.3 | 5.5 |
| ***Other diseases*** | | | | | | |
| *Diabetes* | 1 080 | 20.9 | 978 | 18.9 | 4.9 | 0.6 |
| *Asthma/COPD* | 642 | 12.4 | 573 | 11.1 | 4.1 | 0.9 |
| *Rheumatoid arthritis* | 255 | 4.9 | 225 | 4.4 | 2.8 | 0.8 |
| *Epilepsy* | 442 | 8.5 | 17 | 0.3 | 40.7 | 6.8 |
| *Head trauma* | 361 | 7.0 | 269 | 5.2 | 7.4 | 4.9 |

| | | | | | | |
|---|---|---|---|---|---|---|
| *Hip fracture* | 359 | 7.0 | 270 | 5.2 | 7.2 | 1.2 |

AD, Alzheimer Disease; AED, antiepileptic drug; COPD, chronic obstructive pulmonary disease.

[*] Time from AD diagnosis to the date of antiepileptic initiation of the corresponding user.

[†] Highest occupational social class prior AD diagnosis.

**Table S3. Characteristics of the antiepileptic drug users by type of antiepileptic drug (n=5 137[*]).**

| | Pregabalin (n=2 291) | Valproic acid (n=1 582) | Carbamazepine (n=297) | Clonazepam (n=256) | Oxcarbazepine (n=214) | Gabapentin (n=307) | Phenytoin (n=92) | Other AEDs (n=98) |
|---|---|---|---|---|---|---|---|---|
| Age (years), mean(SD) | 81.9 (6.6) | 79.7 (8.4) | 79.4 (8.5) | 78.6 (7.0) | 78.0 (9.1) | 81.4 (7.2) | 80.8 (7.6) | 79.4 (7.8) |
| Age (years), % | | | | | | | | |
| *< 65* | 1.7 | 6.7 | 6.7 | 3.5 | 10.7 | 2.3 | 4.4 | 4.1 |
| *65–74* | 11.9 | 19.7 | 18.2 | 23.8 | 21.5 | 15.3 | 14.1 | 21.4 |
| *75–84* | 52.2 | 44.9 | 48.5 | 54.7 | 43.0 | 48.5 | 51.1 | 50.0 |
| *≥ 85* | 34.2 | 28.7 | 26.6 | 18.0 | 24.8 | 33.9 | 30.4 | 24.5 |
| Female, % | 72.8 | 63.2 | 59.6 | 59.8 | 60.7 | 64.2 | 65.2 | 65.3 |
| Time since AD diagnosis[†], mean(SD) | 851.8 (725.0) | 1308.1 (843.5) | 1033.1 (746.8) | 841.6 (788.7) | 1033.6 (785.8) | 929.6 (788.0) | 808.8 (686.8) | 1179.3 (831.9) |
| University hospital catchment area, % | | | | | | | | |
| *Helsinki* | 33.6 | 32.3 | 40.1 | 24.6 | 22.9 | 19.2 | 7.6 | 29.6 |
| *Turku* | 12.6 | 13.3 | 7.7 | 17.2 | 17.3 | 13.7 | 20.7 | 16.3 |
| *Tampere* | 24.5 | 16.5 | 10.8 | 18.0 | 22.0 | 19.5 | 32.6 | 19.4 |
| *Kuopio* | 16.2 | 15.0 | 29.3 | 22.3 | 24.8 | 21.8 | 20.7 | 16.3 |
| *Oulu* | 13.1 | 22.9 | 12.1 | 18.0 | 13.1 | 25.7 | 18.5 | 18.4 |
| Occupational status[‡], % | | | | | | | | |

| | | | | | | | | |
|---|---|---|---|---|---|---|---|---|
| *Managerial/professional* | 44.9 | 41.8 | 46.8 | 38.7 | 35.5 | 48.2 | 35.9 | 45.9 |
| *Office* | 15.8 | 15.5 | 14.1 | 18.4 | 18.2 | 21.8 | 26.1 | 9.2 |
| *Farming/forestry* | 8.9 | 9.0 | 5.1 | 7.4 | 10.3 | 6.2 | 8.7 | 9.2 |
| *Sales, industrial, cleaning* | 21.3 | 25.7 | 26.3 | 26.6 | 26.2 | 15.6 | 18.5 | 28.6 |
| *Unknown, no response* | 9.1 | 8.0 | 7.7 | 9.0 | 9.8 | 8.1 | 10.9 | 7.1 |
| **Medication use, %** | | | | | | | | |
| *Antidepressants* | 45.7 | 43.9 | 34.7 | 41.4 | 32.7 | 41.4 | 23.9 | 46.9 |
| *Antipsychotics* | 25.3 | 60.0 | 35.7 | 40.6 | 38.8 | 19.2 | 32.6 | 45.9 |
| *Benzodiazepines* | 47.5 | 45.9 | 34.0 | 47.3 | 36.9 | 37.5 | 42.4 | 41.8 |
| *Antithrombotic agents* | 27.2 | 23.7 | 20.5 | 23.8 | 25.2 | 33.5 | 21.7 | 22.4 |
| *Nonsteroidal anti-inflammatory drugs* | 32.8 | 12.4 | 14.5 | 23.4 | 14.5 | 31.6 | 16.3 | 22.4 |
| **Comorbidities, %** | | | | | | | | |
| ***Cardiovascular diseases*** | | | | | | | | |
| *Hypertension* | 45.7 | 43.8 | 41.4 | 38.7 | 39.2 | 50.2 | 42.4 | 52.0 |
| *Ischemic heart disease* | 31.3 | 24.9 | 26.3 | 32.8 | 27.1 | 38.1 | 22.8 | 32.6 |
| *Chronic heart failure* | 15.7 | 10.3 | 12.5 | 8.2 | 9.8 | 16.3 | 6.5 | 18.4 |
| *Cardiac arrhythmia* | 19.2 | 15.5 | 14.8 | 15.2 | 15.9 | 22.5 | 6.5 | 20.4 |
| *Peripheral vascular disease* | 5.3 | 1.8 | 2.7 | 1.6 | 0.5 | 4.2 | 0 | 3.1 |
| ***Mental diseases*** | | | | | | | | |
| *Schizophrenia* | 0.6 | 0.8 | 0.7 | 0.4 | 1.4 | 0.3 | 0 | 0 |
| *Depression or bipolar disorder* | 6.0 | 5.9 | 5.4 | 9.4 | 3.7 | 4.9 | 3.3 | 11.2 |

| | | | | | | | | |
|---|---|---|---|---|---|---|---|---|
| *Substance abuse* | 2.5 | 3.6 | 5.7 | 3.5 | 3.3 | 2.3 | 2.2 | 7.1 |
| ***Other diseases*** | | | | | | | | |
| *Diabetes* | 23.7 | 17.6 | 16.2 | 16.4 | 12.6 | 31.6 | 21.7 | 21.4 |
| *Asthma/COPD* | 14.5 | 10.7 | 10.1 | 7.8 | 7.0 | 17.9 | 4.3 | 11.2 |
| *Rheumatoid arthritis* | 6.3 | 3.5 | 3.3 | 3.5 | 4.2 | 5.6 | 5.0 | 3.1 |
| *Epilepsy* | 0.8 | 15.5 | 20.2 | 0.8 | 29.4 | 1.3 | 15.2 | 23.5 |
| *Head trauma* | 6.2 | 7.7 | 9.4 | 4.7 | 9.3 | 5.2 | 9.8 | 10.2 |
| *Hip fracture* | 7.2 | 7.5 | 8.1 | 4.3 | 7.5 | 5.9 | 3.3 | 4.1 |

AD, Alzheimer Disease; AED, antiepileptic drug; COPD, chronic obstructive pulmonary disease.

* Users initiating with polypharmacy were excluded (n=30).

† Time from AD diagnosis to the date of antiepileptic initiation.

‡ Highest occupational social class prior to AD diagnosis
